# Green Synthesized of *Thymus vulgaris* Chitosan Nanoparticles Induce Relative WRKY-Genes Expression in *Solanum lycopersicum* against *Fusarium solani*, the Causal Agent of Root Rot Disease

**DOI:** 10.3390/plants11223129

**Published:** 2022-11-16

**Authors:** Sawsan Abd-Ellatif, Amira A. Ibrahim, Fatmah A. Safhi, Elsayed S. Abdel Razik, Sanaa S. A. Kabeil, Salman Aloufi, Amal A. Alyamani, Mostafa M. Basuoni, Salha Mesfer ALshamrani, Hazem S. Elshafie

**Affiliations:** 1 Bioprocess Development Department, Genetic Engineering and Biotechnology Research Institute, City of Scientific Research and Technology Applications, Alexandria 21934, Egypt; 2 Botany and Microbiology Department, Faculty of Science, Arish University, Al-Arish 45511, Egypt; 3 Department of Biology, College of Science, Princess Nourah bint Abdulrahman University, Riyadh 11671, Saudi Arabia; 4 Plant Protection and Biomolecular Diagnosis Department, Arid Lands Cultivation Research Institute, City of Scientific Research and Technology Applications, Alexandria 21934, Egypt; 5 Protein Research Department, Genetic Engineering and Biotechnology Research Institute, City of Scientific Research and Technology Applications, Alexandria 21934, Egypt; 6 Department of Biotechnology, Faculty of Sciences, Taif University, Taif 21944, Saudi Arabia; 7 Botany and Microbiology Department, Faculty of Science (Boys), Al-Azhar University, Cairo 11884, Egypt; 8 Department of Biology, College of Science, University of Jeddah, Jeddah 21493, Saudi Arabia; 9 School of Agricultural, Forestry, Food and Environmental Sciences (SAFE), University of Basilicata, 85100 Potenza, Italy

**Keywords:** WRKY transcription factor, defense-related genes, biotic stress, fusarium root rot, antioxidant enzymes, antifungal activity

## Abstract

*Fusarium solani* is a plant pathogenic fungus that causes tomato root rot disease and yield losses in tomato production. The current study’s main goal is testing the antibacterial efficacy of chitosan nanoparticles loaded with *Thyme vulgaris* essential oil (ThE-CsNPs) against *F. solani* in vitro and in vivo. GC-MS analysis was used to determine the chemical constituents of thyme EO. ThE-CsNPs were investigated using transmission electron microscopy before being physicochemically characterized using FT-IR. ThE-CsNPs were tested for antifungal activity against *F. solani* mycelial growth in vitro. A pot trial was conducted to determine the most effective dose of ThE-CsNPs on the morph/physiological characteristics of *Solanum lycopersicum*, as well as the severity of fusarium root rot. The relative gene expression of WRKY transcript factors and defense-associated genes were quantified in root tissues under all treatment conditions. In vitro results revealed that ThE-CsNPs (1%) had potent antifungal efficacy against *F. solani* radial mycelium growth. The expression of three WRKY transcription factors and three tomato defense-related genes was upregulated. Total phenolic, flavonoid content, and antioxidant enzyme activity were all increased. The outfindings of this study strongly suggested the use of ThE-CsNPs in controlling fusarium root rot on tomatoes; however, other experiments remain necessary before they are recommended.

## 1. Introduction

Tomato (*Solanum lycopersicum* L.) is one of the most widely cultivated vegetable crops worldwide. For those participating in its value chains, the tomato industry provides a significant source of revenue since it is represent a superior source of vitamins, potassium, other minerals, antioxidants and fibers [1,2,3]. A number of biotic and abiotic stress conditions severely limit the growth and economic productivity of tomatoes [4,5]. Numerous soil-borne pathogens of various horticultural and food crops, including *Fusarium* pathogens, cause lethal vascular wilts, rots, and damping-off diseases [6]. Some *Fusarium* species can produce mycotoxins in food and agricultural products, in addition to the losses caused by pre/postharvest losses [7]. *Fusarium oxysporum* f. sp. *lycopersici* (Fol) and *F. solani* (Fol) are two of the most important causes contributing to economic loss in tomato production [8].

Fusarium root rot disease has been found in numerous regions and is rather frequent (25–55%) in tomatoes [9,10]. Under favorable fungal infection weather conditions, the potential economic losses in tomatoes could be enhanced by up to 80% [11]. According to the literature, fungal mycotoxins have a substantial phytotoxic effect, cause pathogenicity, and are required for root rot disease [12]. According to El-Saadony et al. [13,14], the exposure to mycotoxin metabolites may have a moderately damaging effect on animal cells and opportunistic infections in humans.

Nanotechnology is one of the most active fields of research being an effective antimicrobial activity tool [15,16,17,18,19]. Additionally, chitosan treatment controls the number of defense genes in plants, particularly the activation of signaling pathways, which results in the acquisition of plant antitoxin and pathogenesis-related (PR) proteins [20]. Chitosan has been studied to control several pre- and postharvest diseases [21]. *Thymus* sp., belonging to the *Lamiaceae* family, contains over 300 evergreen species that are naturally grown in Southern Europe and Asia. Much research has supported the therapeutic potential of thyme EO in the treatment of cancer, immune deficiency syndrome, infection, and retinal neovascularization [22,23,24].

Gene expression changes cause and occupy these alterations, according to Saijo et al. [25]. It has been proposed that plant transcription factors, which belong to several families, take part in stress reduction or other coping mechanisms when exposed to stress by altering the gene expression patterns. According to Vives-Peris et al. [26], the WRKY transcription factors (Tfs) are a group of plant-specific zinc finger-type regulatory proteins playing a critical role in plant development and the defense response to various abiotic and biotic stresses. The WRKY proteins modulate downstream target genes, other genes (encoding Tfs), or their own expression in order to control the expression of the genes either directly or indirectly [27]. WRKY TFs are essential for controlling a wide range of biological functions, but they are particularly important for controlling how plants react to biotic and abiotic stresses in tomatoes [28,29,30]. Dynamic changes in the quantities of accumulated WRKY transcripts inside the cell can be used to establish how the plant defense regulation involving WRKY proteins is regulated [31]. The main objective of the current research was to determine the most efficient doses of ThE-CsNPs able to inhibit the growth of *F. solani*, causing root rot disease for *S. lycopersicum*. There may be alterations in total phenols, certain antioxidant enzyme activity, and seedling germination investigating the function and mechanism of WRKY family members and their responses to fusarium root rot disease resistance. It will be estimated by examining the expression levels of defense-related genes in tomato roots, such as glucanase A, defensin, and chitinase, as well as transcript factors WRKY4, WRKY31, and WRKY37.

## 2. Results

### 2.1. GC-MS Composition of T. vulgaris EO

The GC-MS analysis results of *T. vulgaris* EO showed bioactive compounds, as listed below in Table 1. In particular, the main constituents are: thymol, α-Pinene, camphene, carvacrol, caryophyllen, carvacrol, myrcene, α-terpineol (Figure S1). The highest percentages of constituents presented in *T. vulgaris* EO were camphene (35.97%), cyclohexane (10.1%), myrcene (7.6%), and α-pinene (6.5%).

### 2.2. Characterization of ThE-CsNPs

#### 2.2.1. Transmission Electron Microscope

**Figure 1:**
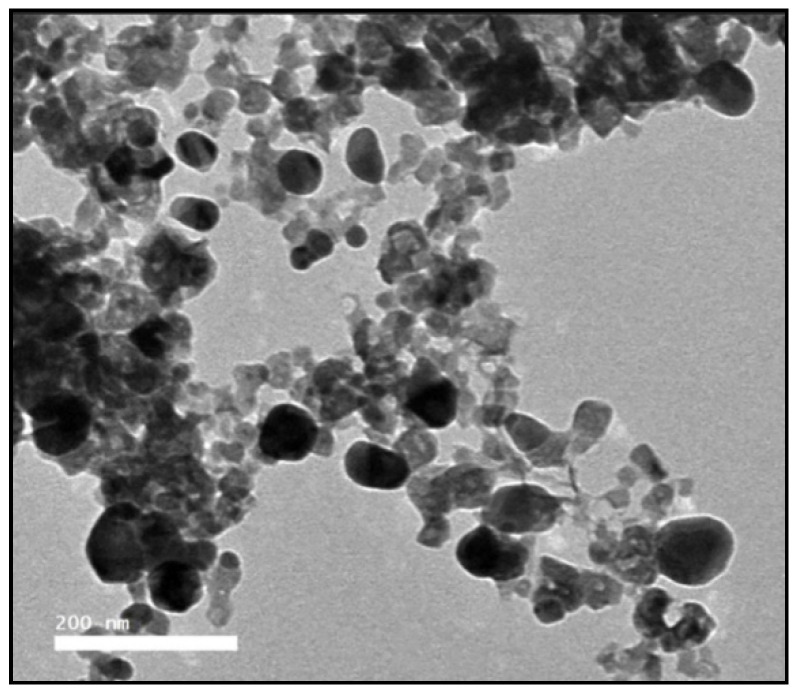
TEM microphotography of ThE-CsNPs.

A vital characterization method for obtaining quantitative estimates of particle size, size distribution, and morphology of nanomaterials is transmission electron microscopy (TEM). The generated ThE-CsNPs by using TEM was illustrated in Figure 1, where the majority of the particles were spherical and irregular. ThE-CsNPs had an average size ranging between 20 and 80 (±0.84) nm.

#### 2.2.2. Fourier Transform Infrared Spectroscopy

The chemical structure of the components involved in ThE-CsNPs was characterized using the Fourier transform infrared (FT-IR) technology, as illustrated in Figure S2, which demonstrates the presence of numerous functional groups in its structure.

### 2.3. In Vitro Antifungal Activity

**Figure 2:**
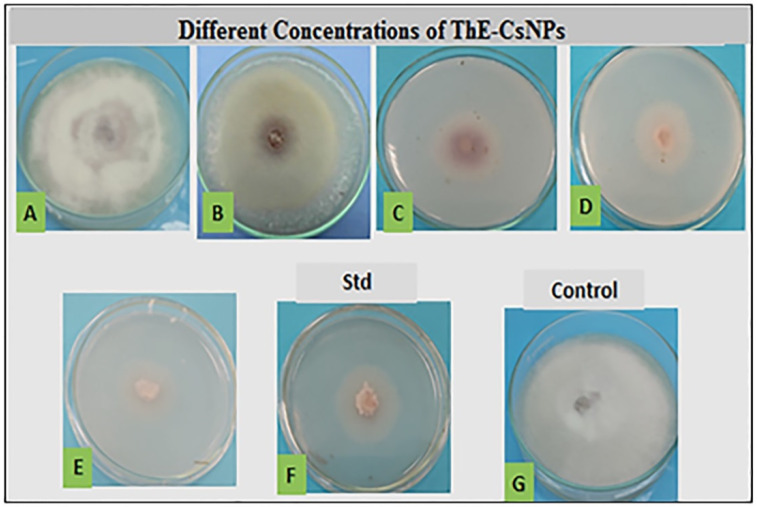
The antifungal activity of ThE-CsNPs at different concentrations against *F. solani*. Where (**A**) 0.2, (**B**) 0.4, (**C**) 0.6, (**D**) 0.8, (**E**) ThE-CsNPs (1%), (**F**) nystatin (0.05%), and (**G**) negative control.

In vitro antifungal activities of different concentrations (0.2, 0.4, 0.6, 0.8, and 1.0%) of ThE-CsNPs were assessed against the *F. solani* pathogen. The average reduction in *F. solani* radial growth in response to treatment is illustrated in Figure 2. All tested concentrations exhibited varied inhibitory activity as compared with the positive control fungicide nystatin (0.05%) and the untreated experimental control. Inhibition of mycelium growth increased with time. After 7 days of incubation at 28 ± 2 °C, the highest growth reduction percentage (89.6 ± 0.0) was observed at ThE-CsNPs (1%) solution treatment, followed by (69.0 ± 0.0 and 31.0 ± 0.0) at 0.8 and 0.6% ThE-CsNPs, respectively, as compared with (62.1 ± 0.1) reduction by using nystatin (0.05%). The minimum growth inhibition (20.8 ± 0.0) was obtained at 0.2% ThE-CsNPs treatment.

### 2.4. In Vivo Trial

#### 2.4.1. Pathogenicity Assay

**Figure 3:**
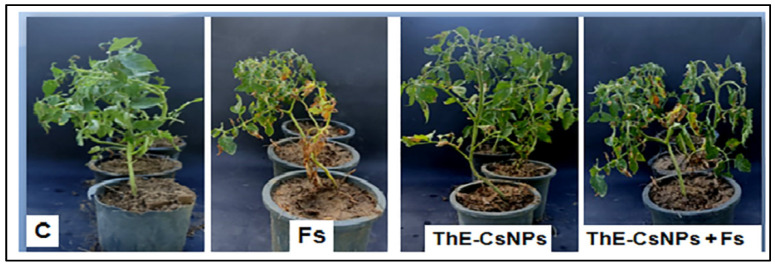
Effect of ThE-CsNPs (1%) on *S. lycopersicum* seedling growth under fusarium root rot disease (20 DAF).

**Figure 4:**
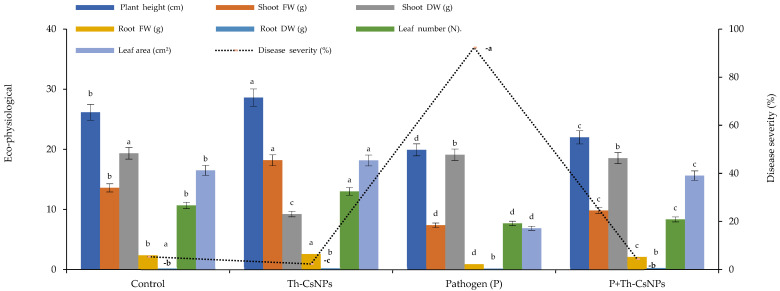
Effects of ThE-CsNPs on disease severity and eco-physiological parameters of *S. lycopersicum* seedlings. Bars with different letters indicate significant differences between treatments at *p* ≤ 0.05. Data are expressed as mean values of three replicates (±SDs).

The first signs of the root rot disease of fusarium appeared 18 days after infection (DAF) (Figure 3). In particular, the highest disease severity (DS) was recorded as 92.4% in case of positive disease control. In contrast, the lowest DS (4.3%) was observed in case of ThE-CsNPs infected seedlings compared to ThE-CsNPs primed seedlings (2.3%) (Figure 4).

#### 2.4.2. Eco-Physiological Parameters

It was found that the priming of tomato seedlings with ThE-CsNPs (1%) resulted in the profuse growth of the tomato plants. Morphological characters differed by using ThE-CsNPs treatment after 20 days; plant height was increased after ThE-CsNPs with a value of 28.6 cm. Shoot dry weights were also increased after ThE-CsNPs treatment to 18.18 g. Root fresh and root dry weights were 2.6 and 0.3 g, respectively after treatment as presented in Figure 4.

#### 2.4.3. Physiological and Biochemical Characteristics

**Figure 5:**
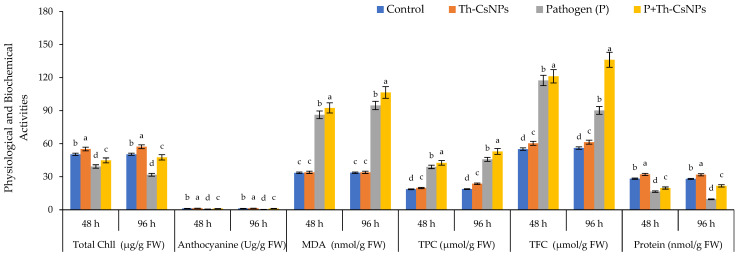
Effects of ThE-CsNPs on total chlorophyll, anthocyanin, MDA, TPC, TFC, and PC level in *S. lycopersicum* seedling under fusarium root rot disease infection. Bars with different letters indicate significant differences between treatments at *p* ≤ 0.05. Data are expressed as mean values of three replicates (±SDs).

Total chlorophyll recorded the highest value at 55.2 and 57.2 µg/g FW in case of using ThE-CsNPs after 48 and 96 h, respectively. Anthocyanin content was 1.3 and 1.4 Ug/g FW after 48 and 96 h, respectively, in case of using ThE-CsNPs. The highest malondialdehyde (MDA) contents (92.4, 106.4) were observed after 48 h and 96 h, respectively, of *F. solani* inoculation under P+Th-CsNPs primed. The total phenolic contents (TPC) were recorded for control, and ThE-CsNPs primed tomato plant under *F. solani* (F.s) infestation condition, and the results showed a gradual increase in TPC level at 48 h and significant elevations at 96 h post inoculation with values 42.6 and 52.9 µmol/g FW, respectively, under P+Th-CsNPs primed. The total flavonoid contents (TFC) were determined in the control and ThE-CsNPs-primed tomato plant under *F. solani* infestation condition, and the results showed significant elevations at 96 h of infestation as compared with control and gradually increased at 48 h post infestation. The highest TFC content was 136.1 µmol/g FW observed after 96 h of *F. solani* inoculation under P+Th-CsNPs primed and at 48 h of *F. solani* inoculation under P+Th-CsNPs primed was 121.1 µmol/g FW. Protein content (PC) in tomato plants was increased under P+Th-CsNPs at 48 and 96 h with values of 19.7 and 21.7 nmol/g FW, respectively, as presented in Figure 5.

#### 2.4.4. Antioxidant Enzymes Activities

**Figure 6:**
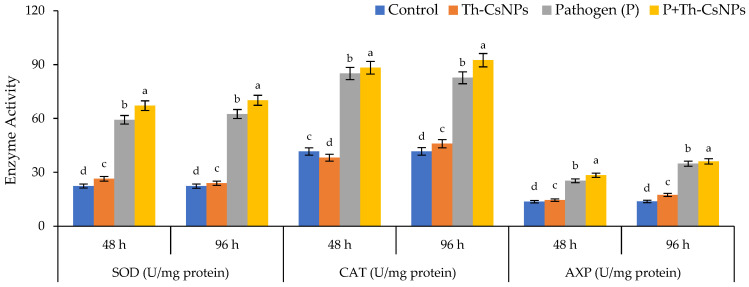
Effects of ThE-CsNPs on SOD, CAT, and AXP enzyme activity in *S. lycopersicum* seedling under fusarium root rot disease infection. Bars with different letters indicate significant differences between treatments at *p* ≤ 0.05. Data are expressed as mean values of three replicates (±SDs).

Data presented in Figure 6 represent the effects of ThE-CsNPs priming on tomato superoxide dismutase (SOD), catalase (CAT), and ascorbate peroxidase (APX) antioxidant enzymes under *F. solani* inoculation at different times intervals (48 and 96 h post fungal inoculation). Data obtained indicated that infection of tomato plants with *F. solani* to an induction in the activities of all studied enzymes when compared with ThE-CsNPs primed and untreated control plants at 48 and 96 h. The highest SOD enzyme activity was 67.1 and 70.2 U/mg protein observed after 48 and 96 h, respectively, under P+ThE-CsNPs primed tomato seedlings. The maximum CAT enzyme activity values 88.3 and 92.5 U/mg were observed after 48 and 96 h of F.s inoculation under P+ThE-CsNPs primed and tomato seedling. The maximum APX enzyme activity values were 28.4 and 36.1 U/mg protein after 48 and 96 h, respectively, of F.s inoculation under P+ThE-CsNPs primed tomato seedlings as compared with the control.

### 2.5. Molecular Analysis

**Figure 7:**
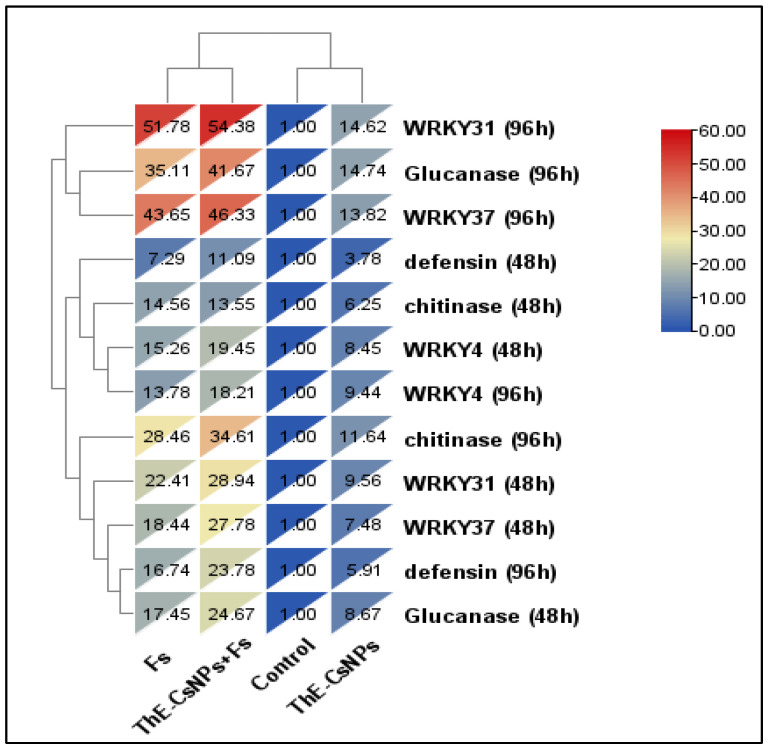
Hierarchical clustering heat map of transcriptional expression of three WRKY transcription factors and some defense-related genes in tomato plant infected with *F. solani* and/or primed with ThE-CsNPs (1%) after 2 and 4 days post *F. solani* inoculation. Where, C: untreated control, Fs: infected with *F. solani*, ThE-CsNPs: thyme extract loaded chitosan nanoparticles solution at 1.0%, and ThE-CsNPs+ Fs: infected tomato plants with *F. solani* and primed with ThE-CsNPs (1%).

With the help of mRNA, qRT-PCR was used to measure the expression levels of the several WRKY transcription factors, which are crucial for both biotic and abiotic tolerance and include WRKY4, WRKY31, WRKY31, and WRKY37. Additionally, chitinase (PR3) and defensin (PR12) genes were represented by qRT-PCR as the relative expression levels of defense-related proteins in tomato plant roots two weeks after *Fusarium* inoculation and after the application of ThE-CsNPs. Hierarchical clustering heat map of transcriptional expression of the investigated WRKYs transcripts factors (WRKY4, WRKY31, and WRKY37) and defense-related (β-1,3-glucanase, defensin, chitinase) genes in tomato roots (Figure 7). As represented from the heat map, all tested treatments are grouped into two main clusters, the first one represents the untreated control plants, and ThE-CsNPs primed plants at 48 and 96 h post *F. solani* inoculation, while the other represents the Fs infected plants that primed with ThE-CsNPs solution or not at 48 and 96 h.

In our study, the highest expression mRNA level (28.9 and 54.4) at 48 and 96 h post inoculation with ThE-CsNPs priming, respectively, was recorded in the WRKY31 gene in roots, followed by (27.8 and 46.3) at 48 and 96 h post inoculation with ThE-CsNPs priming, respectively recorded in WRKY37 gene in roots. Overall, the WRKY TFs genes (WRKY1, WRKY4, WRKY31, and WRKY53) in roots showed positive expression levels (upregulation) under infection.

## 3. Discussion

Producing crops, especially vegetables, can be severely hampered by soil-borne illnesses brought on by a variety of fungi, including *F. solani*. On the remains of the host plant, in the organic matter of the soil, or as free-living organisms, soil-borne infections frequently persist for protracted durations. Even with standard methods, they are frequently challenging to control. In addition, these soil-borne fungi developed resistance to chemical fungicides. Crop productivity can be significantly hampered by soil-borne illnesses brought on by a variety of fungi, including *F. solani*, especially for vegetable crops. On the remains of the host plant, in the soil’s organic matter, or as free-living organisms, soil-borne infections frequently last for a very long time. Even when using traditional techniques, they are frequently challenging to be managed. Therefore, finding innovative, safe, and efficient fungicide solutions to manage plant infections such as tomato root rot is a serious challenge. Since the focus is now on creating non-toxic, safe nanoparticles, the idea of using nanoparticles as an antibacterial agent is relatively new [32].

The thought of using nanoparticles as antifungal agents is relatively new, as the emphasis has since changed toward creating secure, non-toxic nanoparticles [33]. Root rot disease in tomato crops can be controlled in many ways by utilizing chemical and biological approaches [34]. The latest management trend for reducing the negative effects of chemicals (fungicides) is a biological control for pathogenic fungi [35,36]. Microorganisms, semi-chemical products, naturally derived goods derived from plants, and living microorganisms are the four different forms of biocontrol management [37,38,39,40]. 

Additionally, plants essential oils are efficient biocontrol agents against a range of pathogenic bacteria and fungi. Plant EOs are much potential because of their diverse origins and abilities to prevent mycelial growth and spore germination and regulate the vitality of conidia. In comparison to traditional pesticides, nano-emulsions created from these plant EOs may be more effective in controlling pathogens [41,42,43,44]. Researchers assessing the potential phytotoxicity of plant extracts have demonstrated that systemic administration has an impact on Fol [45].

The mode of action of any control agents is crucial and aids in enhancing their efficacy when we encounter efficacy problems. The current study demonstrated in vitro and in vivo effects ThE-CsNPs against root rot disease of tomato infected with *F. solani*. Results of in vitro assay showed that ThE-CsNPs (1%) has greatly inhibited the linear growth of the tested pathogenic fungi. Chitosan changes the rhizosphere’s balance, favoring helpful microbes, including *Bacillus*, *Pseudomonas fluorescens*, *Actinomycetes*, *Mycorrhiza*, and *Rhizobacteria*, while disadvantages microbiological pathogens [46,47]. It has been demonstrated that treating soil with chitin and/or chitosan reduced the rate of nematode infection of plant roots [46,47] and increased the suppressiveness against soil-borne diseases. 

In fact, chitosan/microbe interaction causes a variety of changes in cell permeability as follows: (1) bringing electronegative charges on the outer surface of fungal membrane; (2) addition of polycationic character on the chitosan amino group, interfering with homeostasis ions (K+, Ca^2+^) and, hence, enhancing the outflow of small molecules that interfere with fungal respiration [48]; (3) microbial starvation due to the chelation process between chitosan-metals-vital nutrients; (4) inhibition of the synthesis of mRNA and proteins, which is related to their capacity to pass through the microbe cell membrane and subsequently bind to DNA [48]. The efficiency of chitosan is most frequently attributed to changes in the composition and/or the activity of soil microbiota [49].

Generally, the antimicrobial activity of plant EOs has been verified by several researchers all over the world, where they explained that EOs are able to disrupt the cellular enzyme system and causing the stiffness loss of the hyphal cell wall [50]. Furthermore, the presence of terpenes, alcohols, and phenolic compounds in the composition of EOs are important to improve the physiological and biochemical parameters of tomato seedlings [51,52].

The obtained results confirmed that thyme EO has antifungal effect against fusarium infection at all tested concentrations. In particular, the highest tested concentration (1%) was the lethal dose increased the mycelium growth reduction up to 89.7%. On the other hand, thyme EO has significantly reduced *Botrytis cinerea* colonization on pretreatment detached leaves and significantly decreased the severity of fusarium wilt up to 30.8% particularly 7 days after treatment [53].

According to the chemical analysis, the obtained results showed that the studied thyme EO is a thymol chemotype in agreement with Al-Asmari et al. [54]. In particular, the current study also explicated that the main constituents of thyme EO are camphene (36.1%) and cyclohexane (10.12%). Consequently, Micucci et al. [55] reported that thyme EO is of the thymol type, where carvacrol and p-cymene are the main components. Terpenoids such as thymol and carvacrol are frequently present in the EO and play a significant role in its biological activity [56,57]. The Food and Drug Administration (FDA) has approved them for use in food [57]. Thymol and carvacrol are extremely efficient against foodborne bacteria such as *Salmonella* spp. and *Staphylococcus aureus* [58,59]. The same authors reported that the mechanism of antimicrobial action of this EO could be due to the synergic effect between thymol, citral, and carvacrol, which can transport across microbe membranes [60,61]. In a different study, Isham et al. [62] showed that linalool had an antifungal effect on *Candida albicans* inhibiting the development of germ tubes and biofilm.

Diverse regulatory mechanisms are present in members of the WRKY family. Through the cis-acting mechanism, their protein can be effectively coupled with W-box elements and bind to acting regions to activate or inhibit the transcription of downstream target genes [63]. Thus, WRKY as a transcription factor contributes significantly to plant defense against various pathogen attacks. This reaction either directly or indirectly activates the expression of resistance genes. According to literature review, WRKY DNA binding proteins bind to the promoter region of the plant defense system-activating Arabidopsis natriuretic peptide receptor 1 (NPR1) [64].

The current findings also showed that overexpression of chitinase, defensin, and WRKY transcripts contributing the plant resistance against *F. solani* and reduce its severity. Additionally, pathogen infection substantially elevated WRKY transcripts and PR3 and PR12 genes, which are already regarded as markers for the plant-microbe interaction. Our findings are in agreement with our previous research, which concluded that WRKY3 and WRKY4 encode two structurally related WRKY proteins and that both of these proteins’ expressions were sensitive to stressful environments. Pathogen infection further increased the expression of WRKY4, which was induced by stress but not WRKY3. These findings imply that WRKY4 regulates crosstalk between SA and JA/ET-mediated signaling pathways and, hence, its role in resistance to *F. solani* root rots disease. It is interesting to underline that some WRKY genes, including WRKY4, WRKY33, redundant WRKY18, WRKY40, and WRKY60, contribute to plant resistance to necrotrophic diseases [65,66].

## 4. Materials and Methods

### 4.1. Sample Collection, Identification, and Preparation

The Department of Plant Protection and Biomolecular Diagnosis, Arid Lands Cultivation Research Institute, Egypt, provided the single spore culture (monosporic culture) of *F. solani* (KJ831188) isolate. The *F. solani* isolate was cultivated on potato dextrose agar (PDA) media in plate and slant at 28 °C for 10 days, followed by microscopic analysis with lactophenol cotton blue stain. Up to the next bioassay, the fungus was kept at 4 °C. Tomato seeds (Dania commercial hybrid) variety with suitable appearance and uniform size were purchased from the Egyptian Ministry of Agriculture.

### 4.2. Medicinal Plant Material

On the basis of their ethno-medicinal importance and literature, the *T. vulgaris* plant was collected from an aromatic ornamental garden at Al Nubaria, Egypt (longitudes 30°10′ E and latitudes 30°52′ N) in May 2022. The collected plants were identified and authenticated by the Department of Botany, Faculty of Science—Mansoura University, Egypt, using standard references.

#### 4.2.1. Extractions of Essential Oils

*T. vulgaris* fresh leaves had been previously harvested and sterilized for 30 min with sodium hypochlorite (2%) [67], then were rinsed with sterile distilled water. Fresh leaves had been air-dried, ground, and kept in sealed vials under darkness until use. Hydro-distillation of leaf powders has been conducted for 3 h using a Clevenger-style device.Ltd., India’s New Delhi [68].

#### 4.2.2. GC-MS of Essential Oils

The volatile components of thyme EO have been screened using GC-MS-QP2010 Ultra analysis equipment. The oven temperature schedule started at 50 °C, held for 3 min, then rose by 8 °C/min to 250 °C, holding for 10 min. In electron impact mode, the spectrophotometer was used. The injector, interface, and ion source were maintained at 250, 250, and 220 °C, respectively. Helium served as the carrier gas for the split injection, which used a split ratio of 1:20 and a column flow rate of 1.5 mL/min to inject a 1 µL diluted sample in *n.*hexane (1:1, *v*/*v*). The main single components were identified using WILEY and National Institute of Standards and Technology (NIST08) libraries based on their relative indices and mass spectra.

#### 4.2.3. Preparation of ThE-CsNPs

ThE-CsNPs have been prepared using the ionic gelation process and sodium tripolyphosphate (TPP) as a crosslinking agent as follows. Low molecular weight (LMW) chitosan (0.2 g) at 5% was dissolved in 40 mL of acetic acid (1% *v*/*v*) by stirring at 1000 rpm overnight at room temperature. The pH was then raised to 4.6 by adding 1M NaOH. A 0.45 µm syringe filter was then used to filter the chitosan solution. About 200 µL of *T. vulgaris* EO was added to the CS solution. After that, 8 mL of TPP (2 mg/mL) was added dropwise (1 mL/min) and adjusted to pH = 4.0, with stirring, at 900 rpm for 1 h, then centrifuged at 10,000× *g* rpm for 10 min. The precipitate was collected and oven-dried at 40 °C and kept at room temperature.

#### 4.2.4. Fourier Transform Infrared Analysis

The functional groups of ThE-CsNPs were characterized using FT-IR analysis in order to confirm their synthesis. ThE-CsNPs powder was mixed with potassium bromide (KBr) in a 1:100 ratio, and a Shimadzu FT-IR (Shimadzu 8400S, Kyoto, Japan) apparatus was used to record the spectra.

#### 4.2.5. Transmission Electron Microscopy

The form and size of ThE-CsNPs nanoparticles were investigated using transmission electron microscopy (JEOL JEM-2100 equipment, JEOL Ltd., Tokyo, Japan). The sample was prepared by placing a drop on a copper grid, coating it with carbon, and drying it under a lamp.

### 4.3. In Vitro Antifungal Activity of ThE-CsNPs

The in vitro antifungal efficacy of ThE-CsNPs was carried out in the Bacteriology-Phytopathology Laboratory in the School of Agricultural, Forestry, Food and Environmental Sciences (SAFE), University of Basilicata, Potenza, Italy. The radial mycelia growth of *F. solani* was assessed using the incorporation method [69]. Different concentrations of ThE-CsNPs were used as 0.2, 0.4, 0.6, 0.8 and 1.0% were prepared to determine the most efficient dose with the highest antifungal effect. Nystatin (0.05%) was used as a synthetic (8 μL/150 mL PDA Petri). Sterile distilled water was used as a negative control. About 0.5 cm Ø of *F. solani* fresh culture was inoculated in the center of Petri dishes. The Petri dishes were incubated at 24 °C, then the diameter of mycelium was measured in cm, and the growth reduction percentage (GR %) was calculated using Formula (1). The whole experiment was carried out twice with triplicates.

(1) $$\mathrm{GR}\;(\%)=\frac{\mathrm{Diameter}\;\mathrm{of}\;\mathrm{mycelium}\;\mathrm{in}\;\mathrm{treated}\;\mathrm{plates}\;\left(\mathrm{cm}\right)\;}{\mathrm{Diameter}\;\mathrm{of}\;\mathrm{myclium}\;\mathrm{in}\;\mathrm{contraol}\;\mathrm{plates}\;\left(\mathrm{cm}\right)}\times 100$$

### 4.4. In Vivo Greenhouse Trial

#### 4.4.1. Seeds Treatments

Tomato seeds were surface sterilized in sodium hypochlorite for 30 min, followed by five sterile water washes. In the first experiment, tomato seeds were treated by soaking on 1.0% ThE-CsNPs as a priming solution for 5 h prior to germination in peat moss, while the control treatment was steeped in dH_2_O. Hoagland solution at 1/4 strength was frequently applied to the peat moss.

#### 4.4.2. Pathogenicity Assay

Fungal suspension of *F. solani* (10^5^–10^6^ spores/mL), from 7 days fresh culture, was used for the pathogenicity test for root rot disease. Fresh tomato seedlings (20 d age) were uprooted either from control and ThE-CsNPs-treated ones, washed, surface sterilized with 0.1% mercuric chloride, and immersed in the prepared *F. solani* spore suspension for 60 min. Plants were grown in greenhouses (12 h light/dark and temperature from 18 to 30 °C) and were irrigated daily. The symptoms of root rot disease were first noticed 2–3 weeks after inoculation.

#### 4.4.3. Pot Trials

Four pot groups were arranged in a randomized complete block design with five replications and regularly irrigated with ¼ strength Hoagland solution as necessary and kept under natural daylight and humidity at 65% until the end of the experiment. The four groups were: (1) tomato seedlings were grown under controlled conditions (control, C); (2) inoculated tomato seedlings only with *F. solani* (pathogen, P); (3) the control tomato seedlings foliar-treated with ThE-CsNPs (1%) contains one drop of tween-40 in the surrounding areas of plant stems (C+ ThE-CsNPs); (4) the infected seedlings (15 days after infection) were foliar-treated with ThE-CsNPs (1%) solution (P+ThE-CsNPs). All seedlings were irrigated regularly for 20 days.

#### 4.4.4. Disease Assessments

Disease severity (DS) of fusarium root rot was assessed, 15 days after infection, using the scale described by Filion et al. [70] (Formula (2)).

(2) $$\mathrm{Disease}\;\mathrm{severity}\;(\%)=\frac{\Sigma \mathrm{ab}}{AK}\times 100$$

where: (a) number of diseased plants with the same infection degree; (b) infection degree; (*A*) total number of the assessed plants; and (*K*) the greatest infection degree.

#### 4.4.5. Eco-Physiological Parameters

Sampling was performed 10 to 20 days of treatment with ThE-CsNPs (1%). Morphological traits for both treated and untreated plants were measured. Three plants of each experiment were harvested and transferred to the laboratory and carefully uprooted and were measured for plant height, leaf number, and leaf area. After that, the plants were measured for shoot and root fresh weight and shoot and root dry weight after oven drying at 40 °C for 48 h.

#### 4.4.6. Biochemical Assessment

Malondialdehyde content (MDA) in fresh tomato leaves from treated seedlings with ThE-CsNPs (1%) and control ones were measured following the method described by Heath and Packer [71]. Briefly, 0.5 g fresh tomato leaf was homogenized with 10 mL ethanol and centrifuged at 12,000× *g* for 15 min. After that, 1 mL of supernatant was added to 2 mL of thiobarbituric acid (0.65%) and trichloroacetic acid (20%) mixture. This mixture was boiled for 30 min, cooled rapidly, and centrifuged at 12,000× *g* for 10 min. The MDA content was determined in the supernatant at an absorbance of 532 and 600 nm using a UV-VIS spectrometer (Jenway, Essex, UK). Total phenolic content (TPC) for all treatments was determined by dissolving 5 mg of air-dried powder of leaf in 10 mL methanol using the Folin–Ciocalteu reagent protocol [72]. The total flavonoid content (TFC) for all treatments was measured using the aluminum chloride colorimetry method [73]. The content of soluble protein was estimated for all treatments following Bradford [74].

For total chlorophyll analysis, extracts were produced in triplicate by adding 2 mL of 90% MeOH containing 10% water (*v*/*v*) to 20 mg of each sample, sonicated for 1 h at 4 °C. The crude extract was centrifuged for 20 min at 4000× *g* rpm/4 °C to separate plant debris and supernatant. After that, the supernatant was filtered using a 0.45 m filter, and then 1.5 mL of filtrate was collected for the measurement of functional components. About 1.5 mL of a 10 diluted solution and 150 µL of the 1.5 mL filtered supernatant were combined with 90% MeOH containing 10% H_2_O for analysis. Using a spectrophotometer (Cary 60 UV-Vis, Agilent Technologies, Santa Clara, CA, USA), the absorbance of the diluted sample solution was measured at 665.2, 652.4, and 470.0 nm wavelengths, as described previously [75]. The spectrophotometer’s specifications include a Xenon Flash Lamp (80 Hz) as a light source, measuring wavelengths ranging from 190 to 1100 nm with a resolution of 1.5 nm. Total chlorophyll a (Chla) and total chlorophyll b (Chlb) were calculated using absorbance (A) at each wavelength (Formulas (3) and (4)).

(3) $$\mathrm{Chla}\;(µg\;{\mathrm{mL}}^{-1})=16.82\;A665.2-9.28\;A652.4$$

(4) $$\mathrm{Chlb}\;(µg\;{\mathrm{mL}}^{-1})=36.92\;A652.4-16.54\;A665.2$$

The total anthocyanin analysis has been carried out following the method described by Yang et al. [76] with some minor modifications. The sample extracts were made in triplicate. Briefly, 20 mg of the sample was sonicated for 1 h at 60 °C in 2 mL of acidic MeOH containing 1% HCl (*v*/*v*). The crude extract was centrifuged and filtered in the same manner as samples were pre-processed for total chlorophylls. Then, 300 µL of the 1.5 mL filtered supernatant was diluted with MeOH containing 1% HCl, yielding a 5 diluted solution of 1.5 mL volume. The solution’s absorbance was measured at 530 and 600 nm wavelengths, and the results were used to compute the total anthocyanin concentrations using Formula (5) [76].

(5) $$y\;(µg\;g^{-1})=\left(A530-\;A600\right)\frac{V\;x\;n\;x\;Mw}{Ɛx\;m}$$

where A530 is the absorbance at 530 nm; A600 is the absorbance at 600 nm; *V* is the total volume of the extracted solution; *n* is the dilution ratio; *Mw* is the molecular weight of cyanidin-3-glucoside (i.e., 449.4); Ɛ is the anthocyanin molar extinction coefficient (29.60 M^−1^·cm^−1^); and *m* is the sample mass.

#### 4.4.7. Assay of Antioxidant Enzymes

Antioxidant enzymes were extracted by homogenizing 1 g fresh tomato leaf tissue in chilled 50 mM phosphate buffer (pH 7.0) supplemented with 1% polyvinyl pyrolidine and 1 mM EDTA using a prechilled pestle and mortar. Centrifuging at 18,000× *g* for 30 min at 4 °C, the supernatant was used for enzyme assay.

The activity of superoxide dismutase (SOD, EC 1.15.1.1) and NBT photochemical reductions were recorded at 560 nm in a 1.5 mL assay mixture containing sodium phosphate buffer (50 mM, pH 7.5), 100 μL EDTA, L-methionine, 75 μM NBT, riboflavin, and 100 μL enzyme extract. After 15 min of incubation, the light was switched off, and the activity was expressed as EU mg^−1^ protein.

The catalase activity assay (CAT, EC1.11.1.6) was carried out according to Luck [77], where the change in absorbance was monitored at 240 nm for 2 min, and the extinction coefficient of 39.4 mM^−1^·cm^−1^ was used for the calculation.

Ascorbate peroxidase activity assay (APX, EC 1.11.1.11) was carried out by monitoring absorption change at 290 nm for 3 min in 1 mL reaction mixture containing potassium phosphate buffer (pH 7.0), 0.5 mM ascorbic acid, hydrogen peroxide, and enzyme extract. The calculation of the extinction coefficient of 2.8 mM/cm was used.

### 4.5. Molecular Analysis

#### 4.5.1. RNA Extraction and cDNA Synthesis

The relative expression of distinctly upregulated WRKY transcripts factors and defense-related genes under each treated condition was carried out in the central lab of the Faculty of Science, Arish University (August 2022) and measured quantitatively in root tissues at different time intervals (24 and 48 h) after Fs infestation. Total RNA was isolated from 0.5 g from fresh leaves of tomato plants for each treatment after 10 days from infestation by using the TRIZOL reagent (Invitrogen, Waltham, MA, USA) according to the manufacturer’s protocol. The purified RNA was analyzed on 1% agarose gel. For each sample, to obtain cDNA, 10 µL total RNA was treated with DNAse RNAse-free (Fermentas, Waltham, MA, USA), 5 μL of which was reverse transcribed in a reaction mixture consisting of oligo dT primer (10 pml/μL), 2.5 μL 5× buffer, 2.5 μL MgCl_2_, 2.5 μL 2.5 mM dNTPs, 4 μL from oligo (dT), 0.2 μL (5 unit/μL) reverse transcriptase (Promega, Germany) and 2.5 μL RNA. RT-PCR amplification was performed in a thermal cycler PCR at 42 °C for 1 h and 80 °C for 15 min.

#### 4.5.2. Real-Time Quantitative PCR Analysis

The quantitative study was performed to evaluate the temporal expression of accumulated WRKY transcripts factors and other defense-related genes in all four treatment conditions. Quantitative real-time PCR was carried out on 1 μL diluted cDNA by triplicate using the real-time analysis using (Rotor-Gene 6000, Germany) system, and the primer sequences used in qRT-PCR were given in Table S1. Primers of three defense-related genes (chitinase, defensin, and β-1,3-Glucanase) and three WRKY TFs genes (WRKY4, WRKY31, WRKY37) and housekeeping gene (reference gene) as listed in Table S1 were used for gene expression. Analysis used an SYBR^®^ Green-based method; a total reaction volume of 20 µL was used. The reactions mixture consists of 2 µL of template, 10 µL of SYBR Green Master Mix, 2 µL of reverse primer, 2 µL of forward primer, and sterile distilled water for a total volume of 20 µL. PCR assays were performed using the following conditions: 95 °C for 15 min followed by 40 cycles of 95 °C for 30 s and 58 °C for 30 s. The CT of each sample was used to calculate ΔCT values (target gene CT subtracted from β-Actin gene CT). The relative gene expression was determined using the 2^−ΔΔCT^ method [78].

### 4.6. Statistical Analysis

The obtained data were expressed as the mean ± standard deviation. Statistical analysis was performed using the statistical package SPSS for social sciences (SPSS) 16. All outfindings were analyzed using a one-way ANOVA analysis of variance performed for the significant difference at the *p* < 0.05 level. In contrast, the heatmap was created to compare and contrast the examined treatment responses against different used genes using the TB tools software [79].

## 5. Conclusions

This study concluded that ThE-CsNPs explicated a potential antifungal activity against *F. solani*, the causal agent of root rot disease infecting tomato plants. Additionally, the use of EO showed a promising growth promotion effect for tomato seedlings by enhancing their physiological, biochemical, and antioxidant activities. The three transcription factors: WRKY4, WRKY31, and WRKY37, as well as the other three defense-related genes glucanase A, defensin, and chitinase, have been upregulated due to the effect of ThE-CsNPs (1%). As an overall conclusion, the use of biological fungicides based on *T. vulgaris* EOs with chitosan nanoparticles might be used as a natural alternative to promote tomato growth and control serious phytopathogens.

## Figures and Tables

**Table 1:** The list of main constituents present in *T. vulgaris* EO using GC-MS analysis.

Quantitative ID	Component Identified	Retention Time (min)	Retention Index (RI)	Area (%)	Identification
1	Cyclohexane	4.11	1215	10.12	RI, MS *
2	Myrcene	5.13	106	7.55	RI, MS
3	Caryophyllene	9.14	974	3.25	RI, MS
4	α-Pinene	12.39	993	6.49	RI, MS
5	Camphene	16.81	1429	35.97	RI, MS
6	β-Myrcene	17.74	974	2.77	RI, MS
7	Carene	18.56	1042	2.86	RI, MS
8	p-Cymene	20.35	938	2.19	RI, MS
9	γ-Terpinene	21.16	1062	2.68	RI, MS
10	α-Terpineol	21.53	1138	2.92	RI, MS
11	Linalool	26.13	1126	2.84	RI, MS
12	Thymol	27.85	1074	2.31	RI, MS
13	α-Thymol	29.14	1261	5.17	RI, MS
14	Carvacrol	29.38	1062	2.21	RI, MS
15	Caryophyllen	32.75	1211	4.91	RI, MS
16	Total	-	-	94.24	-

(*) RI: retention index, MS: mass spectroscopy.

## Data Availability

Relevant data applicable to this research are within the paper.

## Supplementary Materials

The following supporting information can be downloaded at: https://www.mdpi.com/article/10.3390/plants11223129/s1, Figure S1: GC-MS profile of *T. vulgaris* EO; Figure S2: FT-IR spectrum of ThE-CsNPs; Table S1: Oligonucleaotide primers used in qRT-PCR analysis.
