# Supplementary material for: Green Synthesized of Thymus vulgaris Chitosan Nanoparticles Induce Relative WRKY-Genes Expression in Solanum lycopersicum against Fusarium solani, the Causal Agent of Root Rot Disease
Source: Plants (Basel). 2022 Nov 16;11(22):3129. doi: 10.3390/plants11223129 (PMC9695361; doi:10.3390/plants11223129)
Supplement: Supplementary file 1 [file plants-11-03129-s001.zip › plants-1980767-supplementary.pdf]

Supplementary Materials

Article

Green Synthesized of *Thymus vulgaris* Chitosan Nanoparticles Induce Relative WRKY-Genes Expression in *Solanum lycopersicum* against *Fusarium solani,* the Causal Agent of Root Rot Disease

Sawsan Abd-Ellatif [1,†], Amira A. Ibrahim [2,†], Fatmah A. Safhi [3], Elsayed S. Abdel Razik [4], Sanaa S. A. Kabeil [5], Salman Aloufi [6], Amal A. Alyamani [6], Mostafa M. Basuoni [7], Salha Mesfer ALshamrani [8] and Hazem S. Elshafie [9,*]

[1] Bioprocess Development Department, Genetic Engineering and Biotechnology Research Institute, City of Scientific Research and Technology Applications, Alexandria 21934, Egypt
[2] Botany and Microbiology Department, Faculty of Science, Arish University, Al-Arish 45511, Egypt
[3] Department of Biology, College of Science, Princess Nourah bint Abdulrahman University, Riyadh 11671, Saudi Arabia
[4] Plant Protection and Biomolecular Diagnosis Department, Arid Lands Cultivation Research Institute, City of Scientific Research and Technology Applications, Alexandria 21934, Egypt
[5] Protein Research Department, Genetic Engineering and Biotechnology Research Institute, City of Scientific Research and Technology Applications, Alexandria 21934, Egypt
[6] Department of Biotechnology, Faculty of Sciences, Taif University, Taif 21944, Saudi Arabia
[7] Botany and Microbiology Department, Faculty of Science (Boys), Al-Azhar University, Cairo 11884, Egypt
[8] Department of Biology, College of Science, University of Jeddah, Jeddah 21493, Saudi Arabia
[9] School of Agricultural, Forestry, Food and Environmental Sciences (SAFE), University of Basilicata, 85100 Potenza, Italy
* Correspondence: hazem.elshafie@unibas.it; Tel.: +39-0971-205522; Fax: +39-0971-205503
† These authors contributed equally to this work.

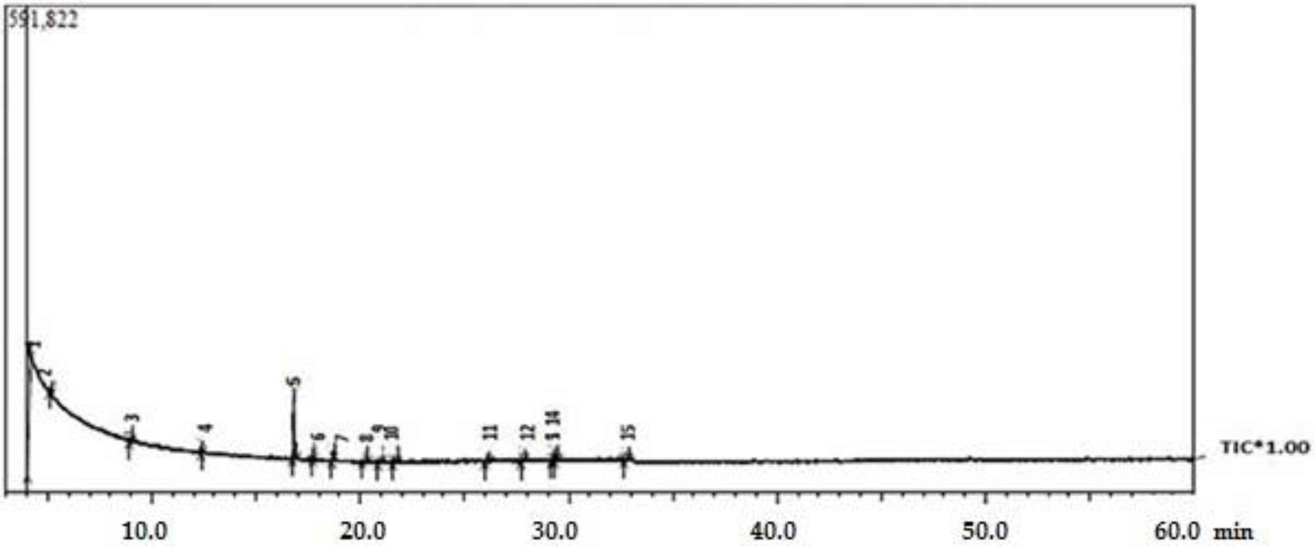

Figure S1. GC-MS profile of T. vulgaris EO.

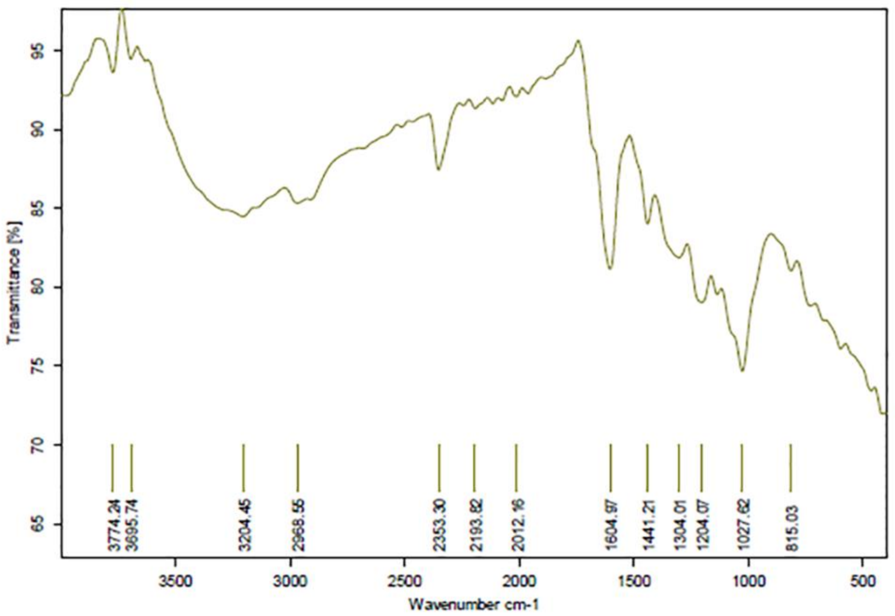

Figure S2. FT-IR spectrum of ThE-CsNPs.

Table S1. Oligonucleaotide primers used in qRT-PCR analysis.

**Gene Name**		**Sequence**	Tm
Chitenase	F	5′-GTCAAGGGGGACCTTGTTTT -3′	58^{0} C
	R	5′CATGTGTGACATGAGCGAAG -3′	
Defensin	F	5′-CCAAATGCCTCGTCATCT-3′	
	R	5′-ATTAGAGTCAAGCTCAAAAGG-3′	
β-1,3-Glucanase	F	5′- AGACAACGTCCGAGGGTATG-3′	
	R	5′- TTTTTCAAGGGCCGAGTATG-3′	
WRKY4	F	5´- CGTTGCACATACCCTGGATG -3’	
	R	5´- GGCCTCCAAGTTGCAATCTC -3’	
WRKY31	F	5′- CCACCTCCTTCACTTCCATT -3′	
	R	5′- GATGGAAAACTCCCAGTCGT -3′	
WRKY37	F	5′- CAGATGCAGCAGTTCAAAGG -3′	
	R	5′- CTTCGAGGGACACATGTTGA -3′	
β-Actin	F	5´-GTGCCCATTTACGAAGGATA- 3´	
	R	5´-GAAGACTCCATGCCGATCAT- 3´	
GAPDH	F	5´- TTGGTTTCCACTGACTTCGTT - 3´	
	R	5´-CTGTAGCCCCACTCGTTGT - 3´	
